# Ashwagandha’s Multifaceted Effects on Human Health: Impact on Vascular Endothelium, Inflammation, Lipid Metabolism, and Cardiovascular Outcomes—A Review

**DOI:** 10.3390/nu16152481

**Published:** 2024-07-31

**Authors:** Michał Wiciński, Anna Fajkiel-Madajczyk, Zuzanna Kurant, Sara Liss, Paweł Szyperski, Monika Szambelan, Bartłomiej Gromadzki, Iga Rupniak, Maciej Słupski, Iwona Sadowska-Krawczenko

**Affiliations:** 1 Department of Pharmacology and Therapeutics, Faculty of Medicine, Collegium Medicum in Bydgoszcz, Nicolaus Copernicus University, M. Curie Skłodowskiej 9, 85-094 Bydgoszcz, Poland; michal.wicinski@cm.umk.pl (M.W.); zuzanna.kurant@cm.umk.pl (Z.K.); sara.liss@cm.umk.pl (S.L.); pawel.szyperski@cm.umk.pl (P.S.); monika.szambelan@cm.umk.pl (M.S.); bartlomiej.gromadzki@cm.umk.pl (B.G.); 2 Department of Neonatology, Ludwik Rydygier Collegium Medicum in Bydgoszcz, Nicolaus Copernicus University in Torun, ul. Ujejskiego 75, 85-168 Bydgoszcz, Poland; iga.rupniak@cm.umk.pl (I.R.); iwonasadowska@cm.umk.pl (I.S.-K.); 3 Department of Hepatobiliary and General Surgery, Faculty of Medicine, Collegium Medicum in Bydgoszcz, Nicolaus Copernicus University, M. Curie Skłodowskiej 9, 85-094 Bydgoszcz, Poland; maciej.slupski@cm.umk.pl

**Keywords:** ashwagandha, lipid metabolism, inflammation, vascular endothelium, cardiovascular outcomes

## Abstract

*Withania somnifera*, commonly known as Ashwagandha, has been popular for many years. Numerous studies have shown that the extract of this plant, due to its wealth of active substances, can induce anti-inflammatory, neuroprotective, immunomodulatory, hepatoprotective, cardioprotective, anti-diabetic, adaptogenic, anti-arthritic, anti-stress, and antimicrobial effects. This review examines the impact of Ashwagandha extract on the vascular endothelium, inflammation, lipid metabolism, and cardiovascular outcomes. Studies have shown that Ashwagandha extracts exhibit an anti-angiogenic effect by inhibiting vascular endothelial growth factor (VEGF)-induced capillary sprouting and formation by lowering the mean density of microvessels. Furthermore, the results of numerous studies highlight the anti-inflammatory role of Ashwagandha extract, as the action of this plant causes a decrease in the expression of pro-inflammatory cytokines. Interestingly, withanolides, present in Ashwagandha root, have shown the ability to inhibit the differentiation of preadipocytes into adipocytes. Research results have also proved that *W. somnifera* demonstrates cardioprotective effects due to its antioxidant properties and reduces ischemia/reperfusion-induced apoptosis. It seems that this plant can be successfully used as a potential treatment for several conditions, mainly those with increased inflammation. More research is needed to elucidate the exact mechanisms by which the substances contained in *W. somnifera* extracts can act in the human body.

## 1. Introduction

The increased risk of developing lifestyle diseases in the global population has led scientists to search for natural products that may have a beneficial effect in treating these conditions. Preparations of plant origin often seem to be safer than synthetic drugs, which can cause dangerous side effects and do not change the progression of some diseases in the long run [1]. Medicinal plant extracts are of great interest as potential drugs and elements of complementary therapies that can reduce the amount of drugs taken by the patient. Moreover, substances of plant origin have multidirectional effects, thanks to which they can be used in preventive treatments, which allows for maintaining appropriate homeostasis in the body. Finally, they can be used as a part of conventional treatment, which requires adequate evidence of their effectiveness [2].

Ashwagandha is the name of *Withania somnifera* in Sanskrit. Other terms for this therapeutic plant are Indian ginseng or winter cherry. *W. somnifera* has been known in traditional medicine (Ayurveda and Unani Systems of Medicine) for over 5000 years. The Latin word “somnifera” means “sleep-inducer”, but the therapeutic effects of this plant have been reported in many different fields of medicine [3,4]. A wide range of treatment indications is found due to the anti-inflammatory, antihypoxic, antiischemic, neuroprotective, immunomodulatory, hepatoprotective, cardioprotective, anti-diabetic, adaptogenic, anti-arthritic, anti-stress, and antimicrobial effects [5,6,7]. Nowadays, Ashwagandha supplementation is popular mainly as an anti-stress solution and is believed to improve overall health and longevity.

All parts of the plant (leaves, flowers, seeds, root) have health potential, but the root is the part that is mostly used medicinally [7]. The major phytochemical components contained in roots are alkaloids (convolamine, convoline, convolidine, convolvine), withanolides (withanolide A, withasomnine, withanosides), sitoindosides (β-sitosterol and d-glycoside), and steroids [8,9]. Withanolides are responsible for the best-documented pharmacological activity [9,10]. Withaferin A, one of the most representative withanolides found in *W. somnifera*, is well known for its anti-inflammatory, antioxidant, immunomodulatory, pro-apoptotic, anti-angiogenesis, and anti-adipogenesis effects [11,12].

This review examines the impact of Ashwagandha extract on the vascular endothelium, inflammation, lipid metabolism, and cardiovascular outcomes. The PubMed and Google Scholar databases were searched using combinations of the following keywords: Ashwagandha, *Withania somnifera*, withanolides, inflammation, lipid metabolism, vascular endothelium, blood pressure, and cardiovascular outcomes. The search mainly included research published in the years 2003–2023.

## 2. Vascular Endothelium

The vascular endothelium, the inner lining of the vascular wall, comprises a single layer of endothelial cells (ECs), providing a barrier between blood and tissues [13]. ECs are physiologically active, participating in various critical processes. These include the regulation of vascular tone, coagulation, platelet adhesion and aggregation, inflammation, immune responses, cell proliferation, and angiogenesis [13,14]. As a result, ECs play a significant role in the pathogenesis of conditions such as cardiovascular diseases [15], diabetes mellitus [14], and cancers [14]. Therefore, this has led to a growing field of research focused on substances that could potentially target ECs for therapeutic purposes. *W. somnifera*, for its wealth of active substances, influences the vascular endothelium through multiple mechanisms.

It is recognized that angiogenesis, the process of forming new capillaries, plays a crucial role in many diseases [16,17]. This process is facilitated by the angiogenic vascular endothelial growth factor (VEGF). VEGF interacts with specific receptors—VEGF-1 and VEGF-2—located on the surface of endothelial cells, leading to the formation of new vessels [18]. Mathur et al. conducted a study investigating the anticancer effects of *W. somnifera*, with a particular focus on its impact on angiogenesis [19]. In the chick-chorioallantoic membrane (CAM) assay, the test group was exposed to 10 ng of VEGF along with extracts or fractions of *W. somnifera* at concentrations of 2.5 ng, 5 ng, and 10 ng. Additionally, subcutaneous gel foam sponges were implanted in mice using higher concentrations (100 ng of VEGF in combination with 100 ng of the plant extract) to measure the density and number of vessels formed. After 12 days of in vitro incubation and 14 days of follow-up in mice, the results indicated that *W. somnifera*, particularly at higher concentrations (10 ng in vitro and 100 ng in vivo), inhibited VEGF-induced capillary sprouting and formation by lowering the mean microvessel density (number of blood vessels per unit area), thereby reinforcing Ashwagandha’s role as an anti-angiogenic agent [19].

The ubiquitin–proteasome pathway (UPP) is one of the main pathways that selectively degrades cellular proteins and regulates most processes essential for maintaining cellular function balance [20]. This process involves the covalent attachment of ubiquitin to a substrate protein, forming a polyubiquitin chain (ubiquitylation), which signals the proteolysis of this protein with the involvement of proteasomes [21]. One of the proteins degraded by the UPP is the hypoxia-inducible factor (HIF), which partially promotes new blood vessel formation through the transcription of factors such as VEGF [22]. Another example is the degradation and phosphorylation of nuclear factor kappa B (IκBα), leading to the activation of nuclear factor κB (NF-κB) and its subsequent role in angiogenesis, the cell cycle, apoptosis, carcinogenesis, and immune responses [23,24]. NF-κB activation occurs in response to various factors, including pro-inflammatory cytokines, with tumor necrosis factor-alpha (TNF-α) being the most extensively studied [25]. Mohan et al. carried out a study investigating the effect of *W. somnifera* on these pathways [26]. In their research, human umbilical vein endothelial cells (HUVECs) were induced by growth factors (FGF-2 at 30 ng/mL) and then treated with various concentrations of Ashwagandha fractions (5, 10, and 50 µg/mL). After 24 h of coincubation, the length and quantity of vessels were reduced. This study also found out that *W. somnifera*, at various concentrations (0.2, 1, 5 µM), inhibited NF-κB activation in TNF-α (10 ng/mL)-stimulated HUVECs, as measured by the electrophoretic mobility shift assay (EMSA). Additionally, there was an increase in the level of polyubiquitinated proteins, suggesting the involvement of the UPP in the process of NF-κB inhibition [26].

Further research on Ashwagandha’s role in endothelial-cell-mediated angiogenesis was conducted by Bargagna-Mohan et al. [27]. They pre-treated human choroidal endothelial cells (HCECs) and HUVECs with different concentrations of Ashwagandha extract (0.25, 0.5, 1 µM) for 30 min and then stimulated with TNF-α (10 ng/mL) for 20 min. The study discovered that *W. somnifera* in a dose-dependent manner inhibited the induction of inflammatory IκBα degradation and increased the levels of ubiquitinated species, targeting the UPP and suppressing angiogenic sprouting in an in vitro tube formation assay. Moreover, the use of *W. somnifera* resulted in the induction of the expression of the antioxidant enzyme heme oxygenase-1 (HO-1) in ECs, providing cytoprotective effects and contributing to maintaining vascular homeostasis [27].

Nitric oxide (NO) is an important compound produced in ECs by the constitutive calcium-dependent enzyme nitric oxide synthase (NOS) [28]. There are three NOS isoforms—endothelial NOS (eNOS), neuronal NOS (nNOS), and inducible NOS (iNOS) [29]. NO exerts a potent vasodilating and anti-inflammatory effect, contributing to the maintenance of vascular homeostasis [30]. In a study using rat aortic rings and a human endothelial cell line, Pathak et al. presented a significant increase in NO production mediated by *W. somnifera* (more specifically two extracts from Ashwagandha—standardized root extract of the plant NMITLI-118R (NM) and withanolide A) with the highest NO generation at a concentration of 50 µg/mL for the NM extract and 5 µg/mL for the withanolide A extract [29]. Research has shown that the increase in NO production is mediated through upregulating the expression of eNOS genes and proteins, as measured by Real-Time PCR. NO-induced vasorelaxation is achieved by activating guanylyl cyclase, an enzyme in vascular smooth muscle cells that converts guanosine-5’-triphosphate (GTP) into cyclic guanosine monophosphate (cGMP). This research underscores Ashwagandha’s vasoprotective potential [29].

Furthermore, Iuvone et al. explored the plant’s immunostimulating properties [31]. They examined the impact of *W. somnifera* on NO synthesis in mouse macrophages by stimulating them with various concentrations (1–256 µg/mL) of the Ashwagandha extract. After 24 h, they measured the nitrite levels in the culture and found a significant, concentration-dependent increase in NO production starting from 4 µg/mL of the extract. They also observed the upregulation of iNOS, one of the NOS isoforms, typically synthesized by cells in response to inflammation, possibly mediated through NF-κB transactivation [31].

Endothelial cell dysfunction is triggered by an imbalance between antioxidants and reactive oxygen species (ROS) [32]. This endothelial activation eventually results in a decreased bioavailability of NO, impaired vascular tone [32], initiation of EC apoptosis, and alteration in their angiogenic potential [33]. Khalil et al. carried out a study on rats where induced myocardial infarction caused oxidative stress by generating free radicals [34]. This led to a reduction in the levels of antioxidant enzymes—specifically superoxide dismutase (SOD) and glutathione peroxidase (GPx)—which are responsible for scavenging free radicals. The study showed that treating the animals with 100 mg/kg of *W. somnifera* for 4 weeks enhanced the activity of these enzymes, thereby bolstering the endogenous antioxidant system [34]. A similar study was previously conducted by Kaur et al. on rats with induced pulmonary hypertension and right ventricular hypertrophy [35]. They demonstrated that *W. somnifera*, administered for 5 weeks in doses of 50 and 100 mg/kg, alleviates oxidative stress (more effectively at higher concentrations) by reducing ROS levels. This is achieved through an enhancement in endogenous antioxidant enzymes such as SOD, thereby improving the function of the pulmonary vascular endothelium [35]. Moreover, the effects of Ashwagandha have been associated with an increase in eNOS expression in the lungs of rats, which elevates NO levels and exhibits vasodilatory, antiproliferative, and apoptosis-inducing effects [35]. The results of this study also outlined the anti-inflammatory role of Ashwagandha extract, as the plant’s action resulted in a decrease in the level of expression of pro-inflammatory cytokines TNF-α and NF-κB and an increase in the level of the anti-inflammatory cytokine interleukin-10 (IL-10) [35].

However, not all research findings are consistent with the aforementioned data. For instance, in the study by Kim et al., AGS human gastric epithelial cell lines infected with *Helicobacter pylori* were examined for, among others, VEGF production and HIF-1α levels in the absence or presence of one of the compounds of *W. somnifera*—withaferin A (10–500 nM). The results showed that pre-treatment, as well as co-treatment with withaferin A, did not inhibit the basal or *H. pylori*-induced VEGF production and did not affect HIF-1α stabilization in gastric epithelial cells [36].

Additionally, not every component of *W. somnifera* exhibits the same effect on the vascular endothelium. An example is the study by Chaudhary et al., which compared the properties of withaferin A with its natural and structurally similar analog—2,3-dihydro-3β-methoxy-withaferin A (3βmWi-A). This study demonstrated that in cancer cells, withaferin A reduced the level of VEGF, whereas 3βmWi-A did not have this property. Thereby, the authors of the study suggested that 3βmWi-A does not exhibit anti-metastatic potential [37].

In the studies discussed in this chapter, *W. somnifera* was mainly used as a standalone therapeutic agent, so it can be ruled out that the results were due to a more complex treatment regimen. Despite the promising research results, the referenced studies show that to assess the role and effects of *W. somnifera*, it is essential to conduct more studies that would provide a definitive position on the impact of Ashwagandha on the vascular endothelium. Most of the conducted studies are in vitro or in vivo in rats. There is a lack of typical clinical trials that would allow for the prediction of clinical applications in specific human diseases associated with vascular endothelial dysfunction.

In summary, *W. somnifera* exerts multiple effects on the vascular endothelium. By inhibiting VEGF, preventing NF-κB activation, modulating NO production, inducing antioxidant enzymes like HO-1, SOD, and GPx, and reducing ROS levels, it demonstrates anti-angiogenic, vasodilatory, and anti-inflammatory properties. These findings suggest that Ashwagandha may enhance vascular endothelial function, potentially expanding its medical applications by leveraging its mentioned properties. Table 1 shows a summary of the research discussed in this chapter.

## 3. Inflammation

Chronic inflammation underlies various diseases, including cardiovascular disorders, neurodegenerative conditions, and immune dysfunctions. Also aging, despite the lack of coexistence of diseases, is now considered to be the result of low-grade inflammation. The term “inflammaging” was created, describing the deteriorating organ dysfunction with age [38]. Key pro-inflammatory cytokines like TNF-α and interleukin-1 (IL-1) exacerbate these conditions, while anti-inflammatory cytokines such as IL-10 and transforming growth factor beta (TGF-β) provide a counterbalance [39].

The influence of the external environment, as well as internal processes such as telomere attrition and genome alterations, are factors that modulate inflammation and aging [40]. A wide range of studies have shown that extracts of *W. somnifera* have many properties which modulate multiple pathways of the immune system in rats [41], cats [42], and humans [43].

Immunoglobulin E (IgE) recognizes the foreign antigen and informs mast cells and basophils about it. Subsequent exposure to the same antigen induces a type 2 allergic reaction aimed at activating T helper 2 (Th2) and B lymphocytes and consecutively producing IgE, group 2 innate lymphoid cells (ILC2), eosinophils, and elevated Th2 cytokines—interleukins (IL-4, IL-5, IL-13) [44]. Clinical symptoms of an excessively activated immune system in this pathway include asthma, skin rash, excessive mucus production, and pollen or food allergies. *W. somnifera* has properties that attenuate type 2 allergic reactions by a reduction in cytokines IL-4, IL-13, TNF-α, and IgE [45,46].

Many of the substances contained in Ashwagandha extract have immunomodulatory effects [47]. Withanolides, which are steroid derivatives, influence the hypothalamic–pituitary–adrenal (HPA) axis. Lopresti et al. carried out a double-blind randomized controlled trial (RCT) in which patients took a 240 mg extract of *W. somnifera* with 84 mg of withanolide glycosides. They showed a reduction in morning cortisol and dehydroepiandrosterone (DHEA) after 15 days of use. Clinically, this was reflected in improved mood and reduced anxiety, which was measured using the HAM-A (Hamilton Anxiety Rating Scale) and DASS-21 (Depression Anxiety Stress Scale-21) compared to the placebo group [48]. A reduction in cortisol levels, as well as stress as measured using the Perceived Stress Scale (PSS) and HAM-A depending on the dose of *W. somnifera* extract, was shown in an 8-week study by Salve J. et al. The researchers divided the participants into groups: the first group was given a placebo, the second 250 mg, and the third a 600 mg extract of Ashwagandha. An improvement in emotional state and a reduction in cortisol levels were seen in both groups receiving the root extract, but the higher dose had a better effect [49].

On the one hand, withanolides diminish the level of cortisol, which has an anti-inflammatory effect, and on the other hand, selectively block cyclooxygenase-2 (COX2) and inhibit lipopolysaccharide (LPS)-induced inflammation [50]. Withaferin A inhibits NF-κB, activator protein 1 (AP1), and alpha-2 macroglobulin [51]. Withaferin A in combination with withanolide E can silence the proliferation of B and T lymphocytes and affect the recognition of antigens [26]. The modulation of T lymphocyte activity is based on the effect of withaferin on blocking the activity of Zap70 kinase. The potential advantage could be the prevention of autoimmune-mediated pathologies [52].

Singh et al. showed that the extract from *W. somnifera* root in add-on therapy influences the improvement in forced expiratory volume in one second—FEV1% (increased by 14.36% after 12 weeks of therapy); quality of life; and exercise tolerance in GOLD 2 and 3 categories of chronic obstructive pulmonary disease (COPD) patients. It was proved that withanolides are the most potent in inhibiting the activity of angiotensin-converting enzyme 2 (ACE2), myeloperoxidase (MPO), and interleukin-6 (IL-6), which significantly enhances lung function. Simultaneous anti-inflammatory and free radical lowering effects alleviate obstructive symptoms [53].

The adaptogenic function of *W. somnifera* was proved in influencing transcription RNA, resulting in the regulation of cellular metabolism and the maintenance of homeostasis. Advanced glycation end products (AGEs) are secreted by microglia, and they are a marker of aging and degenerative processes in the brain. They induce iNOS expression and bind to RAGE in neurons, which leads to cells damage and apoptosis. AGEs cause the augmentation of the formation of the amyloid-beta precursor protein (APP) and its derivative amyloid-beta Aβ and tau protein. They create toxins for neuron oligomers. Aβ similarly to AGEs binds to RAGE and activates the extracellular-signal-regulated kinase (ERK) 1/2 pathway and further NF-κB. The effect of this is an increase in the expression of COX2, interleukin-1β (IL-1β), or iNOS. All these factors intensify neuroinflammation [54]. The ability of Ashwagandha to inhibit at a transcriptional level the biosynthesis of Aβ and IL-1β leads to the suppression of neurodegeneration [55].

Panossian et al. proved the ability of *W. somnifera* to downregulate the expression of arachidonate 12-lipoxygenase (ALOX12), leukotriene C4 synthase, and dipeptidase 2 (DPEP2) genes in human neuroglia cells. This leads to the inhibition of neuroinflammation and neurodegeneration [56].

Extended inflammation contributes to chronic organ dysfunction. Cytokines circulating in the blood and those synthesized by the epithelium of the renal tubes make the kidneys extremely sensitive to inflammation. In the kidneys, angiotensin II and TNFα stimulate the signaling pathway of NF-κB [57,58]. The result is the increased expression of genes C-C Motif Chemokine Ligand 2 (CCL2) and C-C Motif Chemokine Ligand 5 (CCL5) and further renal fibrosis. The progressive remodeling of kidneys by increasing fibrosis leads to their loss of function. *W. somnifera* by the downregulation of TNF-α, CCL2, and CCL5 protects the kidneys. A study of the effects of various herbal extracts on rat kidney NRK-52E cells showed that a formulation containing 250 mg of Ashwagandha (including 2.5% withanolides) at a dilution of 1:100 after 24 h of incubation prevents TNF-α- and LPS-induced CCL5 gene expression [59].

The anti-inflammatory and antioxidant properties of Ashwagandha were proven in the liver. *W. somnifera* extract rich in the withanolide fraction reduces the expression of COX-2, iNOS, IL-1β, and TNFα. This was proved by Devkar et al. in a study with acetaminophen-treated rats. The hepatoprotective effect of the withanolide-rich extract was significant and dose-dependent. The doses used in the study were 50 mg/kg, 100 mg/kg, and 200 mg/kg. The downregulation of TNF-α and IL-1β mRNA expression in a dose-dependent manner was observed. iNOS and COX-2 mRNA expressions were reduced only when a 200 mg/kg dose was used [60].

Kaileh et al. in their study focused on the discovery of the mechanisms of action of Ashwagandha and showed using cell cultures (murine fibrosarcoma cells, human kidney cells, IKK-α- and IKK-β-deficient mouse embryonic fibroblasts, cervix cancer cells, human breast cancer cells) that the pre-treatment of the leaf extract of *W. somnifera* blocks the TNF effect by the inhibition of the IκB kinase (IKK) complex. The task of IKK is to phosphorylate IκB inhibitor proteins, under the influence of pro-inflammatory factors, and then release NF-κB. The blockade of this reaction by Ashwagandha prevents NF-κB from entering the cell nucleus and attaching to DNA [61].

An indispensable element of the human organism’s homeostasis is coping with harmful external factors, inflammation, destructive processes, or aging. *W. somnifera* is an adaptogen that helps to adjust and survive the negative effects of damage at the gene expression level. Improving the functioning of the cells, and secondarily of organs, inhibits the effects of aging and extends life. Table 2 shows a summary of the research discussed in this chapter.

## 4. Lipid Metabolism Disorders

Lipid metabolism disorders encompass a range of conditions characterized by abnormalities in the body’s synthesis, breakdown, and transport of lipids. These disorders can lead to elevated levels of cholesterol and triglycerides in the blood, contributing to serious health issues such as cardiovascular disease, diabetes, and metabolic syndrome. Common lipid metabolism disorders include hyperlipidemia, atherosclerosis, and obesity [62,63]. Hyperlipidemia refers to elevated levels of lipids in the blood, which can predispose individuals to atherosclerosis. In this condition, plaque builds up in the arterial walls, leading to reduced blood flow and an increased risk of heart attacks and strokes [64]. Obesity, defined by excessive fat accumulation, further exacerbates these risks and is often associated with insulin resistance and type 2 diabetes [65].

The management of lipid metabolism disorders traditionally involves lifestyle modifications such as diet and exercise and, in many cases, pharmacological treatment. Examples of substances used in treatment are statins or fibrates [64,66]. However, these treatments may not be effective for all patients and sometimes can come with significant side effects. This has spurred interest in complementary therapies, including the use of medicinal plants. Among these, *W. somnifera* has emerged as a promising candidate due to its multiple therapeutic properties.

A pivotal study identified anti-adipogenic withanolides from the root of *W. somnifera*, demonstrating their ability to inhibit the differentiation of preadipocytes into adipocytes [10]. This anti-adipogenic effect is significant as it prevents the formation of new fat cells, playing a crucial role in preventing and managing obesity—a major risk factor for lipid metabolism disorders. In the study, they used 25 µM of withanolides [10]. Further research has shown that *W. somnifera* extract can enhance energy expenditure by improving mitochondrial function in adipose tissue and skeletal muscle [67]. Enhanced mitochondrial activity leads to a higher metabolic rate and increased energy expenditure, which helps reduce body fat and improve lipid profiles. These findings underscore the herb’s potential in addressing metabolic syndrome, often associated with lipid metabolism disorders.

Another study on Nile tilapia found that dietary supplementation with *W. somnifera* improved the fish’s lipid profile and intestinal histomorphology [68]. Additionally, the study observed a modulation of cytokine responses to *Streptococcus iniae* infection, suggesting an overall enhancement in health and immune response. It is important to point out that this research was performed on fish. However, the implications for human health are still noteworthy, indicating that *W. somnifera* could also enhance human lipid metabolism and immune function.

Withaferin A, a bioactive compound in Ashwagandha, modulated oxidative damage by regulating inflammatory mediators and apoptosis through the phosphatidylinositol 3-kinase/protein kinase B (PI3K/AKT) signaling pathway in high-cholesterol-induced atherosclerosis in experimental rats [69]. By reducing oxidative stress and inflammation, withaferin A helps to mitigate the progression of atherosclerosis and other lipid-related disorders. This modulation of oxidative damage is crucial for the management of lipid metabolism disorders, as oxidative stress and inflammation are key contributors to their pathogenesis. In another significant study, withaferin A demonstrated protective effects against high-fat-diet-induced obesity by attenuating oxidative stress, inflammation, and insulin resistance [70]. This protective action is vital for preventing obesity and associated lipid metabolism disorders. The attenuation of oxidative stress and inflammation, coupled with improved insulin sensitivity, highlights the comprehensive benefits of *W. somnifera* in managing high-fat-diet-related health issues.

Research on withanolide A, another compound from *W. somnifera*, showed cytoprotective effects against 7-ketocholesterol-induced cytotoxicity in human brain endothelial cells [71]. This protection is essential for maintaining brain health, especially in conditions where lipid metabolism disorders might lead to neurological complications. By safeguarding brain endothelial cells from lipid-induced damage, withanolide A supports overall metabolic health and highlights the broad therapeutic potential of Ashwagandha.

A review of traditional knowledge and recent research findings has consolidated evidence supporting the role of Ashwagandha in managing metabolic syndrome [72]. This review emphasizes the herb’s efficacy in improving various metabolic parameters, including lipid metabolism, offering a holistic approach to treating metabolic disorders. The integration of traditional knowledge with contemporary scientific research can pave the way for novel therapeutic strategies. Additionally, new anti-adipogenic withanolides, such as withasomniferol D, have been identified from the root of Ashwagandha [73]. These newly discovered compounds expand the potential therapeutic applications of *W. somnifera* in lipid metabolism disorders, presenting new opportunities for research and development. The study investigated withanolides at a concentration of 25 µM.

Lastly, an in vitro study demonstrated the hypolipidemic effects of Ashwagandha and Arjuna, showing a significant reduction in lipid levels [74]. This supports the traditional use of these herbs in managing hyperlipidemia and underscores the importance of integrating traditional practices with modern scientific validation.

The accumulating evidence highlights *W. somnifera*’s multifaceted role in managing lipid metabolism disorders. Its anti-adipogenic properties, enhancement in energy expenditure, improvement in lipid profiles, and protective effects against oxidative stress and inflammation make it a promising candidate for further research and clinical application. Integrating traditional knowledge with modern scientific insights can foster the development of innovative therapeutic strategies for lipid metabolism disorders, offering hope for improved management and better health outcomes. Unfortunately, there is a lack of data on clinical trials testing the potential use of *W. somnifera* in diseases related to lipid metabolism, which should be taken into consideration when using extracts of this plant to improve the organism’s lipid metabolism.

## 5. Cardiovascular Outcomes

According to the WHO (World Health Organization), cardiovascular diseases are the leading cause of death worldwide—they cause about 17.9 deaths each year [75]. The challenge this poses to the world keeps us looking for ways to reduce this statistic.

It is already a well-known fact that blood pressure (BP) is closely related to cardiovascular risk and also to renal function, organs of vision, or endothelial epithelium damage, as we mentioned before in this article [76,77,78]. Therefore, the limit of normal values of BP was determined to be able to start the treatment of hypertension early enough to prevent the consequences caused by its too-high values and to reduce the cardiovascular risk. We decided to review the articles to find out whether *W. somnifera* becoming more and more popular and its active compounds could be used in hypertension treatment and how its properties relate to cardiovascular risk. Does it have any potential?

First, it is important to raise the issue that several studies confirm the anti-anxiety and anti-stress effects of Ashwagandha [79,80,81,82,83]. It is known that prolonged stress is related to hypertension development and thus bears the risk of cardiovascular events [84,85]. The question is as follows: could we draw such a far-reaching conclusion that Ashwagandha, by reducing stress, has a long-term protective effect on vessels and blood pressure and can reduce cardiovascular risk?

In a randomized, double-blind, placebo-controlled trial scientists investigated the efficacy and safety of Ashwagandha root extract in adults experiencing high stress and fatigue. The authors divided 120 overweight or mildly obese women and men feeling stressed and fatigued into two groups—60 of them received the placebo, and the remaining 60 received 200 mg of *W. somnifera* root extract standardized to 1.5% total withanolides, twice daily, for 12 weeks [79]. It turned out that despite a reduction in perceived stress over time, Ashwagandha did not significantly affect the blood pressure among responders.

Our question is, since the effects of hypertension occur in the long term, should the trial and observation not take longer? In fact, should we not address how long-term stress reduction from *W. somnifera* extract supplementation affects the overall cardiovascular risk?

The results of this study seem to be similar to those of another, in which 40 males and 40 females took 300 mg of Ashwagandha or placebo, for 8 weeks, twice daily, orally. According to the scientists, there were no significant changes between these two groups when it comes to body weight, body temperature, pulse rate, respiratory rate, and most interesting to us—systolic (SBP) and diastolic (DBP) BP. The measurements were collected at the baseline and after 8 weeks of supplementation. So, another study does not confirm the effect of Ashwagandha on BP nor on factors related to cardiovascular risk, such as weight [86].

However, existing research suggests that Ashwagandha may influence BP. In a further study, researchers investigated the effect of *W. somnifera* on a group of 51 people with hypertension. The first group received 2 g of Ashwagandha root powder with milk, and the second group received the same substance but with water. Before supplementation, in both groups, the mean SBP was, respectively, 164 mmHg and 157 mmHg, and after supplementation, this decreased to 158 mmHg in the first group and 154 mmHg in the second group. The difference was not significant. Surprisingly, the mean DBP after supplementation with Ashwagandha root extract decreased in the first group from 100.5 mmHg to 85 mmHg and in the second from 101.2 mmHg to 92 mmHg [87]. It seems that the hypotensive effect after *W. somnifera* supplementation is significant and more visible when it comes to DBP than SBP. Moreover, Ashwagandha root extract supplemented with milk is more effective in decreasing BP in comparison to its supplementation with water. To explain the effect of *W. somnifera* action, the authors refer to other studies. They propose that this contributes to reducing stress by *W. somnifera*, which in turn decreases activating HPA and oxidative stress, having a cardioprotective function in this way [87]. Regarding this, we maintain our earlier consideration that there may be a correlation between the stress-reducing effects of Ashwagandha and a potential reduced risk of developing cardiovascular disease, but this requires further research.

The above-mentioned results are quite different to the conclusions from another randomized, controlled, parallel-group, single-blinded study, where a group of healthy college-going young adults between 18 and 25 years old were supplemented with 500 mg capsules of *W. somnifera* extract, once daily, for 8 weeks [88]. According to the authors, in the present study, after the administration of Ashwagandha, the maximum oxygen consumption capacity increased by 6.8% at a moderate intensity, but no significant change was observed in the balance and resting BP. What is interesting in the same study was another study group in which participants were supplemented with *Terminalia arjuna*—it seems that this plant is effective in resting SBP reduction, but what is more, the biggest resting SBP reduction was observed in a group supplemented simultaneously with *W. somnifera* and *Terminalia arjuna* extract [88]. Unfortunately, a few limitations can be noted—the authors did not describe the used dosage of extracts, and more research is needed to investigate which active substances cause these effects. Are these substances contained in *Terminalia arjuna*, or in *W. somnifera*? Maybe the active substances from both of these plants interact and thus lower BP?

The studies discussed above show that Ashwagandha’s effect on BP is questionable, and more research should be conducted to find out how it actually works in this area. Based on the current state of knowledge, we must conclude that *W. somnifera*’s effect on BP is doubtful. More research is needed.

Undeterred by these findings, we went a step further to find out if *W. somnifera* could be used in patients with the lethal manifestation of cardiovascular disease which myocardial infarction potentially is. It turns out that in isoprenaline (synthetic, cardiotoxic catecholamine)-induced myocardial infarction (MI) in rats, Ashwagandha gives cardioprotective effects. The authors investigated how the markers of myocardial damage were changing under the influence of Ashwagandha administration [89]. For a better assessment, they take into account the correlation between biochemical parameters (such as lipid peroxidation product malondialdehyde, endogenous antioxidants such as glutathione, antioxidant enzymes superoxide dismutase, catalase, and glutathione peroxidase, and myocardial enzymes creatinine phosphokinase and lactate dehydrogenase), functional parameters (such as mean arterial pressure, heart rate, left ventricular peak positive pressure change, left ventricular rate of peak negative pressure change, and elevated left ventricular end-diastolic pressure), and histopathological parameters in the examined hearts. It turns out that isoprenaline-induced MI caused a significant decrease in antioxidant enzymes. Interestingly, in the group treated with *W. somnifera*, the level of some antioxidant enzymes increased [89]. These data indicate that Ashwagandha has a protective effect on the myocardium by counteracting the destructive oxidative stress induced by ischemia. However, when it comes to hemodynamic parameters, as in previous studies, Ashwagandha did not improve blood pressure recordings significantly as compared to the isoprenaline control group. It also had no influence on the heart rate, but it has to be mentioned that it reduced the left ventricular end-diastolic pressure, and the myocardial relaxation (left ventricular pressure decline) was notably restored as compared to the isoprenaline control. It also improved contractility. The effects were dose-dependent.

Finishing, the authors referred to histopathological parameters and remarked that Ashwagandha did not significantly prevent myofiber loss but significantly prevented myonecrosis, as indicated by a significant reduction in the infiltration of inflammatory cells and vascular changes as well as edema as compared to the isoprenaline control group [89].

To consolidate the above knowledge, we will refer to the next study, which confirms the cardioprotective effect of *W. somnifera*. The authors divided rats into three groups—a saline control group (sham group) and an ischemia and reperfusion group (control group) where on the 31st day rats were given 45 min of left anterior descending (LAD) coronary artery ligation and 60 min of reperfusion-induced myocardial injury [90]. In the last group, the rats were administered Ashwagandha extract in a dose of 50 mg/kg, for 30 days, as an independent treatment of ischemia. On the 31st day, these rats were also given 45 min of LAD coronary artery ligation and 60 min of reperfusion-induced myocardial injury. The conclusion was drawn that *W. somnifera* significantly reduced lipid peroxidation expressed by a significant reduction in thiobarbituric acid reactive substance (TBARS) levels and restored the creatinine phosphokinase (CPK) in comparison to the control group. When it comes to the histological results, in a sham group, the myocardium had an organized pattern as a normal architecture. In rat hearts subjected to ischemia and reperfusion, the authors observed edema, myonecrosis, inflammation, and myofiber loss. In contrast to this, in a group of pre-treated Ashwagandha rats subjected to ischemia and reperfusion, the occasional focal myofiber loss, necrosis, edema, and inflammation were significantly less as compared to the control group. To summarize the immunohistochemical results, it has to be distinguished that in the Ashwagandha-treated group, the Bax (proapoptotic protein) expression was attenuated in comparison to in the control group (the results of which, in turn, showed an increase in Bax expression compared to the sham group), which suggests that *W. somnifera* may inhibit the apoptosis in injured myocardium. Pre-treatment with Ashwagandha was also associated with greater Bcl-2 expression, which is in turn an anti-apoptotic protein, in comparison to the control group [90].

The results from extensive studies present that *W. somnifera* shows cardioprotective effects due to its antioxidant properties and reduced ischemic/reperfusion-provoked apoptosis, which was confirmed biochemically, histologically, and immunohistochemically.

Another study examined the effect of Ashwagandha on ischemic/reperfused myocytes (MI/R). The subject of the research was primary neonatal cardiomyocytes (NRVMs) isolated from 1- to 2-day-old Sprague Dawley rats administered withaferin A. The apoptotic death was induced by simulated ischemia/reperfusion (SI/R) and hydrogen peroxide (H_2_O_2_) exposure (as a model of oxidative stress). The administration of withaferin A in cells under stress conditions—both SI/R and H_2_O_2_ exposure—caused increased lactate dehydrogenase (LDH) release (as an intracellular enzyme and marker of cell breakdown and apoptosis). When it comes to the molecular mechanism, the authors propose that withaferin A modulates oxidative stress/apoptosis by inducing Akt expression, which is a kinase upregulating peroxiredoxins (Prdx-1), and SOD2 and SOD3 enzymes involved in antioxidant signaling pathways. This study allows major conclusions to be drawn—oxidative stress is the cause of cell death as a result of ischemia and reperfusion, which can be prevented by administering withaferin A which modulates antioxidant processes [91].

It is also worth mentioning a study examining the influence of withaferin A on MI/R injury in wild-type and AMP-activated protein kinase domain-negative (AMPK-DN) gene transgenic mice with reduced infarct size and improved cardiac function. The authors believe that the mechanism behind this effect is the decreased activation of caspase 9 (involved in an intrinsic apoptotic way), upregulating AMP-activated protein kinase (AMPK) phosphorylation and increasing the MI/R-inhibited ratio of Bcl2/Bax. It turned out that in AMPK-DN gene transgenic mice, withaferin A administration did not reduce cardiac dysfunction and infarct size nor did it restore the Bcl2/Bax ratio, which only proves that the cardioprotective mechanism of this substance’s action in the ischemic myocardium is based on the upregulation of the anti-apoptotic mitochondrial pathway in an AMPK-dependent manner [12].

These studies proved that Ashwagandha and its active compounds via its upregulation of the anti-apoptotic mitochondrial pathway and antioxidative action play a cardioprotective role. It cannot be ignored that withaferin A is more effective in the case of smaller doses in both of the research works.

In the studies discussed above, the perceived limitations include the lack of descriptions of the active substances responsible for specific effects, the lack of standardization of doses, and the limited number of clinical trials conducted on the protective effects of Ashwagandha on the cardiovascular system. It seems that *W. somnifera* may have potential cardioprotective properties, but more research is needed to clarify the mechanisms of its action and the reasons for the discrepancies in the above-described studies. Table 3 shows a summary of the research discussed in this chapter.

## 6. Limitations

Despite the promising results of the studies discussed in the chapters above, there are several limitations worth considering. Data from large clinical trials examining Ashwagandha as a medicine are still lacking. It seems reasonable to ask whether Ashwagandha extracts can be used as a standalone treatment for a specific condition or rather as a complementary therapy—an adjuvant to conventional treatment. It is also worth mentioning that most preparations with this plant extract are dietary supplements, which is associated with an easier registration procedure for such products [92].

Ashwagandha root extract is widely regarded as safe and well tolerated [48,86]. On the other hand, it is important to note that plant extracts can have many side effects and contraindications [93]. Recent studies have shown that Ashwagandha is a factor in herbal-induced liver injury (HILI). Ashwagandha-induced HILI manifests as cholestatic hepatitis. In addition, it can lead to acute and chronic liver failure syndrome, which is associated with a high mortality rate. There is an increased risk in people with pre-existing liver disease [94]. In patients with hyperthyroidism, *W. somnifera* extracts can cause symptoms such as irritability, restlessness, nervousness, hand tremors, palpitations, psychomotor agitation, muscle fatigue, and reduced libido [3]. Due to the enhancement in testosterone production by Ashwagandha, men with hormone-sensitive prostate cancer should avoid its use, as it intensifies the progression of the disease [7]. Furthermore, for women planning pregnancies, the use of higher doses of *W. somnifera* root extract can cause miscarriages [7]. Moreover, Ashwagandha has additive effects with anticonvulsants, anti-anxiety medications, and antidepressants, which may lead to a dangerous increase in their side effects [95]. Therefore, despite the relative safety of this plant preparation, it is important to educate patients and medical staff whenever such products are recommended. Importantly, patients’ self-administration of Ashwagandha runs the risk of not controlling other therapies they receive [3].

Another important issue regarding *W. somnifera* is the standardization of the raw material and the assessment of the bioavailability of the active substances from these preparations [3]. The studies discussed above often lacked the standardization of the extracts to a specific content of active substances; hence, the interpretation of the results and comparison between studies are challenging. Some studies have shown that the active compounds contained in Ashwagandha extracts, e.g., withaferin A, have low bioavailability. This fact results in a lower therapeutic effect caused by these substances. Therefore, an important issue seems to be the development of an appropriate formulation of potential drugs, which poses a major challenge for subsequent researchers [3,96]. An additional challenge is that *W. somnifera* is a plant consisting of various components with differing properties. This creates a broad scope for further research in this area to enable the use of *W. somnifera* for disease prevention or treatment.

## 7. Conclusions

This review of the research presented above shows the wide range of effects of Ashwagandha extract on metabolism. Several substances in it (alkaloids, steroids, and probably the most important withanolides) influence various metabolic pathways, thus causing various effects and possibly modifying the course of diseases.

Ashwagandha has a broad effect on the endothelium. It shows an anti-angiogenic impact by inhibiting VEGF-induced capillary sprouting and formation by lowering the mean microvessel density. Also, it causes a significant increase in NO production and alleviates oxidative stress by reducing reactive oxygen species levels.

The text above discusses research on the multidirectional impact of *W. somnifera* on the immune system, producing an anti-inflammatory effect. Ashwagandha has properties that attenuate type 2 allergic reactions by a reduction in cytokines IL-4, IL-13, TNF-α, and IgE, reduction in cortisol levels, as well as stress, the selective blocking of COX2 and inhibition of LPS-induced inflammation, and the inhibition of the activity of ACE2, MPO, and IL-6. Anti-inflammatory and antioxidant properties were proven in the liver and kidney. Additionally, the adaptogenic function of *W. somnifera* was proved to influence transcription RNA, resulting in the regulation of cellular metabolism and maintenance of homeostasis.

Ashwagandha extract also affects lipid metabolism. It inhibits the differentiation of preadipocytes into adipocytes, can enhance energy expenditure by improving mitochondrial function in adipose tissue and skeletal muscle, and shows a significant reduction in lipid levels.

These results suggest that Ashwagandha has many benefits. By exhibiting the above-mentioned effects, this plant can be expected to have a major impact on cardiovascular disease and thus improve cardiovascular outcomes. However, the implications for human health are still noteworthy. There are discrepancies in the scientific research performed.

On the one hand, Ashwagandha did not affect significantly blood pressure. The effect on BP is questionable, and more research should be conducted. There is no significant effect on body weight, body temperature, pulse rate, or respiratory rate. On the other hand, Ashwagandha has a protective effect on the myocardium by counteracting the destructive oxidative stress induced by ischemia. Cardioprotective effects (due to its antioxidant properties and reduced ischemic/reperfusion-provoked apoptosis) have been biochemically, histologically, and immunohistochemically proven.

It seems that *W. somnifera* may have potential mainly anti-inflammatory effects and hence cardioprotective, immunomodulatory, neuroprotective, hepatoprotective, anti-diabetic, adaptogenic, anti-arthritic, and anti-stress effects, but it is still unknown to what extent it has a real impact on the course of diseases. More research is needed to explain the mechanism of action of the substances contained in Ashwagandha to clarify the reasons for the discrepancies in the above-described studies and, most importantly, what real impact they have on patients’ health.

## Figures and Tables

**Table 1:** Summary of reviewed research.

Authors	Subject of Study	Dose	Results
Kaur et al. (2015) [35]	MCT-challenged rats with PH (pulmonary hypertension)	*W. somnifera* root powder (50 and 100 mg/kg/d, p.o.)	↓ RVP, ↓ RVH; ↑ TUNEL-positive cells, ↓ procaspase-3; ↑ IL-10, ↓ TNF-α, ↓ NF-κB; ↑ eNOS, ↓ HIF-1α
Khalil et al. (2015) [34]	Wistar albino rats (*n* = 40)	WSLEt (100 mg/kg) for 4 weeks	↓ heart weight, ↓ cTnI; ↓ TC, ↓ TGs, ↓ VLDL-C, ↑ HDL-C; ↑ SOD, ↑ GRx, ↑ GPx, ↑ GST, ↓ LPO; ↓ inflammatory cells
Iuvone et al. (2003) [31]	The monocyte/macrophage cell line J774	WS (1–256 μg/mL)	↑ NO; ↓ NO synthase inhibitor L-NAME; ↓ TLCK—an inhibitor of NF-κB activation
Mathur et al. (2006) [19]	Chick-chorioallantoic membrane (CAM) with VEGF	2.5, 5, and 10 ng of WS root extract and fractions	↓ mean microvessel density; ↓ MVD
	Subcutaneous implantation of gel foam sponges with VEGF in male Swiss albino mice (25–35 g)	100 ng of WS root extract and fractions	
Mohan et al. (2004) [26]	Human umbilical vein endothelial cells induced by FGF-2	5, 10, and 50 µg/mL of WS fractions; 24 h of coincubation	↓ sprouting index
	Human umbilical vein endothelial cells stimulated with TNF-α	0.2, 1, and 5 µM of withaferin A; 30 min of treatment + 20 min of TNF-α coincubation	↓ TNF-α-induced NF-κB activation; ↑ polyubiquitinated proteins
Bargagna-Mohan et al. (2006) [27]	Human choroidal endothelial cells and human umbilical vein endothelial cells, both stimulated with TNF-α	0.25, 0.5, and 1 µM of withanolide D; 30 min of treatment + 20 min of TNF-α coincubation	↑ IκBα; ↑ ubiquitinated species
	Human choroidal endothelial cells and human umbilical vein endothelial cells, both stimulated with VEGF	0.5, 1, and 2 µM of withaferin A; 12 h	↑ HO-1
Pathak et al. (2017) [29]	Transverse aortic rings (4 mm) of 10-week-old male Wistar rats (250 g)	0.1–100 µg/mL of standardized WS root extract (NM) and 0.1–100 µg/mL of marker compound withanolide A	↑ vasorelaxation
	Human endothelial cell line EA.hy926	3 h of treatment with 0.5–100 µg/mL of NM and 0.5–50 µg/mL of withanolide A	↑ NO; ↑ eNOS
Kim et al. (2015) [36]	AGS cells infected with *H. pylori* in the absence or presence of withaferin A (pre-treated and co-treated)	10–500 nM of withaferin A; 24 h of experiment	↔ VEGF
Chaudhary et al. (2019) [37]	AGS cells pre-treated with withaferin A and infected with *H. pylori* Osteosarcoma cell lines treated with WA and 3βmWi-A	500 nM of withaferin A; 6 h of experiment; 0.3 and 0.6 µM of withaferin A and 3βmWi-A for 48 h	↔ HIF-1α; ↓ VEGF (for withaferin A); ↑ VEGF (for 3βmWi-A)

Note: ↓ reduction, ↑ increase, and ↔ irrelevant; abbreviations: RVP—right ventricle pressure; RVH—right ventricle hypertrophy; cTnI—cardiac troponin I; TC—total cholesterol; TGs—triglycerides; HDL-C—high-density lipoprotein cholesterol; VLDL-C—very-low-density lipoprotein cholesterol; TLCK—trypsin-like serine protease inhibitor; MVD—mitral valve disease; WS—*W. somnifera*.

**Table 2:** Summary of reviewed research.

Authors	Subject of Study	Dose	Results
Lopresti et al. (2019) [48]	Stressed, healthy adults	240 mg of a standardized Ashwagandha extract (Shoden)	↓ morning serum cortisol and ↓ DHEA
Salve et al. (2019) [49]	Stressed healthy adults	250 mg and 600 mg of Ashwagandha extract	↓ morning serum cortisol; 600 mg better effect
Fazil et al. (2021) [52]	T cells	0.3–1.25 μM withaferin A	Inhibition of the ZAP70 kinase and retardation of T-cell motility
Singh et al. 2022 [53]	COPD patients qualified as GOLD 2 and 3	250 mg of WS root capsules	↓ ACE-2, ↓ MPO, and ↓ IL-6
Atluri et al. (2020) [55]	SH-APP cells	50 nM–1 μM of withaferin A	↓ Aβ, ↓ IL-1β, and ↓ NF-κB
Panossian et al. (2018) [56]	Cultivated neuroglial cells	WS (5.0 µg/mL) corresponding dose in humans 300 mg; WSL (1.5 µg/mL) corresponding dose in humans 90 mg	↓ ALOX12, ↓ DPEP2, and ↓ leukotriene C4 synthetase
Grunz-Borgmann et al. (2015) [59]	Rat kidney NRK-52E cell line	450 mg of a standardized extract containing a minimum of 2.5% total withanolides	Inhibition of TNFα on CCL2 and CCL5 gene expression
Devkar et al. (2016) [60]	Male Swiss albino mice	50 mg/kg, 100 mg/kg, and 200 mg/kg of the withanolide-rich extract	Every dose: ↓ TNFα and ↓ IL-1β mRNA expression; 200 mg/kg: ↓ iNOS and ↓ COX-2 mRNA expression
Kaileh et al. (2007) [61]	Murine fibrosarcoma L929sA cells and human embryonic kidney 293T cells, IKK-α- and IKK-β-deficient mouse embryonic fibroblasts and cervix cancer cells (HeLa), and MDA-MB-231 human breast cancer cells	Withaferin A, withanolide A, and 12-deoxywithastramonolide (1 mg/mL)	↓ IL-6; ↓ NFκB B-driven gene expression; ↑ AP1-driven gene expression; ↓ NFκB/DNA binding; ↓ NFκB translocation; ↓ TNF-induced phosphorylation and degradation of IκBα; ↓ TNF-induced IKKβ activity; ↑ induces the phosphorylation of IKKβ through the MEK/ERK Pathway

Note: ↓ reduction, ↑ increase; abbreviations: MEK/ERK—mitogen-activated protein kinase/extracellular-signal-regulated kinase; WS—*W. somnifera*.

**Table 3:** Summary of reviewed research.

Authors	Subject of Study	Dose	Results
Smith et al. (2023) [79]	120 overweight or mildly obese women and men	200 mg of WS root extract standardized to 1.5% total withanolides, twice daily, for 12 weeks	↓ stress; No significant change in BP
Verma et al. (2021) [86]	Randomized, double-blind, placebo-controlled, and parallel-group study; 80 healthy participants	Ashwagandha root extract 300 mg for 8 weeks	No AE reported; ↔ BW, BP; ↔ ALT, AST, ALP; ↔ TSH, fT3, fT4
Kushwaha et al. (2012) [87]	51 stress-oriented hypertensive subjects in the age group of 40 to 70 years old	2 g Ashwagandha root powder, orally, for 91 days (with milk or with water)	↔ BMI; ↔ SBP; ↓ DBP
Sandhu et al. (2010) [88]	Healthy college-going young adults between 18 and 25 years old	500 mg capsules of WS extract (no information about used dosage in capsules) once daily, for 8 weeks	↑ maximum oxygen consumption capacity at moderate intensity; No significant change was observed in balance and resting BP; ↓ resting SBP when supplemented simultaneously with WS and *Terminalia arjuna* extract
Mohanty et al. (2004) [89]	Wistar albino male rats	25, 50, and 100 mg/kg orally for 4 weeks	↑ glutathione (50 and 100 mg/kg); ↑ antioxidant enzyme glutathione peroxidase; ↑ superoxide dismutase; ↑ lactate dehydrogenase; ↑ creatinine phosphokinase; ↔ blood pressure; ↓ left ventricular end-diastolic pressure; ↑ myocardial relaxation (left ventricular pressure decline); ↑ contractility (50 mg/kg); ↓ myonecrosis and ↓ edema
Mohanty et al. (2008) [90]	Adult male Wistar rats	Hydro-alcoholic extract of WS (50 mg/kg) orally, for 30 days	↑ GSH, ↓ TBARS, ↑ CPK; ↓ Bax protein, ↑ Bcl-2; ↓ TUNEL-positive cells
Langade et al. (2019) [83]	Randomized, double-blind, placebo-controlled study of 60 patients with insomnia	Ashwagandha root extract, 300 mg	↓ SOL, ↓ WASO; ↑ TST, ↑ TIB, ↑ SE; ↓ PSQI; ↓ HAM-A
Yan et al. (2018) [91]	Primary neonatal cardiomyocytes (NRVMs) were isolated from 1- to 2-day-old Sprague Dawley rats; 8–10-week-old wild-type mice	fWFA (0 nM, 100 nM, 1000 nM)	↓ apoptotic cell death; ↑ HO-1, ↑ Prdx-1, ↑ SOD-2 (via activation of Akt pathway); ↓ ROS
Guo et al. (2019) [12]	Adult male wild-type (WT) mice and adult male AMPK-DN mice [dominant negative α2-subunit (D157A) of AMPK]	Low-dose (1 mg/kg) or high-dose (5 mg/kg) WFA	(1 mg/kg) ↑ LVEF, ↑ dP/dtmax and dP/dtmin, ↓ infarct size; (5 mg/kg) ↓ dP/dtmax and dP/dtmin (both) ↓ TUNEL staining, ↓ caspase-3 activity; ↑ Bcl2, ↓ Bcl2/ Bax; ↑ AMPK

Note: ↓ reduction, ↑ increase, and ↔ irrelevant; abbreviations: BW—body weight; ALT—alanine transaminase; AST—aspartate transaminase; ALP—alkaline phosphatase; TSH—thyroid-stimulating hormone; fT3—free triiodothyronine; fT4—free thyroxine; SOL—sleep onset latency; WASO—wage after sleep onset; TST—total sleep time; TIB—total time in bed; SE—sleep efficiency; PSQI—Pittsburgh Sleep Quality Index; LVEF—left ventricular ejection fraction; GSH—glutathione; WS—*W. somnifera*.
